# An Etiology Report for Burns Caused by Korean Folk Remedies

**DOI:** 10.1055/a-2040-0826

**Published:** 2023-05-29

**Authors:** Hong Sil Joo, Hyun Been Kim

**Affiliations:** 1 Department of Plastic and Reconstructive Surgery, Hanil General Hospital, Seoul, Korea

**Keywords:** burns, folk remedies, glacial acid, moxibustion

## Abstract

**Background** In this modern era of science-based medicine, some people still accept folk remedies as an alternative form of medicine. However, misinformation and misuse of folk medicines can result in dangerous complications. Among the possible complications of folk remedy use, this study focused on the clinical characteristics of burns caused by folk remedies.

**Methods** We retrospectively reviewed the clinical records of patients who had been treated for burns caused by folk remedies from May 2015 to April 2022. Data were collected on patients' age and gender, type of folk remedy, reason for using the folk remedy, the severity of the burn, the number of wounds, lesion type, and type of treatment.

**Results** We found 59 patients with burns due to folk remedies. Most were female (76.3%) and ≥ 60 years old (72.9%). The most common type of folk remedy was moxibustion (74.6%), followed by the use of glacial acetic acid (20.3%). The reasons for using folk remedies were arthralgia relief (39%), health improvement (18.6%), and treatment of tinea pedis (11.9%). Most patients had multiple wound sites and had burns that were considered severe, requiring surgical treatment (72.9%). The majority of lesions were on the lower extremity, including the foot.

**Conclusion** This study described the risk of burns caused by folk remedies and the clinical characteristics of the wounds. The results emphasize the need for greater public awareness of the risk of burn injuries when using folk remedies.

## Introduction

Folk remedies are medicines or practices handed down from generation to generation based on cultural beliefs and traditions. In 2008, the World Health Organization defined traditional medicine as “the sum total of the knowledge, skills, and practices based on the theories, beliefs, and experiences indigenous to different cultures, whether explicable or not, used in the maintenance of health as well as in the prevention, diagnosis, improvement or treatment of physical and mental illness.” For centuries, until the development of modern medicine, people determined that certain methods were effective for certain diseases based on a variety of experiences. Although most folk treatments have no scientific basis or have been proven to be ineffective, some folk remedies that are based on long-term empirical results are also recognized by modern medicine, and are still used by the public.

Koreans often rely heavily on traditional medicine, which has become even more popular with the development of online information. Although access to directions for the use of folk remedies can be easy to find online, they often do not inform the searcher of the risks or of the scientific data verifying or refuting the remedy's effectiveness.

We researched the cases of patients who were burned because of folk remedy use. Among the complications caused by folk remedies, burn complications were analyzed. This study described the clinical characteristics of burn wounds, the patients' characteristics, and the types of folk remedies that can cause burns. The results of this research may bring awareness to the public of the risks of folk remedies. In addition, descriptions of these burns could help facilitate accurate recognition of folk remedy injuries as well as their proper treatment.

## Methods

We retrospectively reviewed the medical records of patients who were treated for burns due to the use of folk remedies from March 2015 to April 2022. Data were extracted on patients' age and gender, type of folk remedy used, the reason for using the folk remedy (i.e., “for what purpose did you undergo the folk remedy?”), the severity of the burn, the number of wound sites (i.e., single site or multifocal sites), the type of burn lesion, and the type of treatment.

In the case of multiple wounds, the most severe lesion was used as the standard.

Data were summarized and described using univariate analysis and values were presented as number (%).

### Burn Treatment Process

The burn wounds were classified by experienced surgeons. Superficial second-degree burns (edematous and pink wound bed), and deep second-degree burns (mottled or pale white wound bed) were healed by secondary intention. The wounds were irrigated daily. Antibacterial ointment and growth factor-containing ointment were then applied to the wounds and covered with a foam dressing material for absorption of discharge. For third-degree burns (eschar or dry wound bed) or burns that might take more than 3 weeks to heal, surgical treatment such as a skin graft or local flap was recommended.

The requirement for informed consent was waived. The study was approved by the local ethics committee of Hanil General Hospital, Seoul, South Korea (approval no: HGH-2022–08–008).

## Results

There were 59 patients with burn injuries caused by using folk remedies. The patients' details are shown in Table 1 (age, gender, reason for using remedies). Women accounted for 76.3% of all patients (45 female, 14 male). The patients ranged in age from their 20s to 80s, with the majority of patients over 60 years (72.9%), including 33.9% in their 70s. The most common reason for using folk remedies was arthralgia relief (39%), followed by health improvement (18.6%) and cure of tinea pedis (11.9%).

**Table 1:** General etiology of patients

Characteristics	*N* (%)
Sex	
Female	45 (76.3)
Male	14 (23.7)
Age	
20s	3
30s	1
40s	1
50s	9
60s	14 (23.7)
70s	20 (33.9)
80s	9
Reason	
Arthritis (joint pain)	22 (39)
Health improvement	11 (18.6)
Treatment of tinea pedis	7 (11.9)
Abdominal discomfort	6
Removal of pigment lesion (solar lentigo, nevus)	5
Paralysis care	5
Treatment of wart	1
Removal of calluses	1
Fatigue	1

**Fig. 1:**
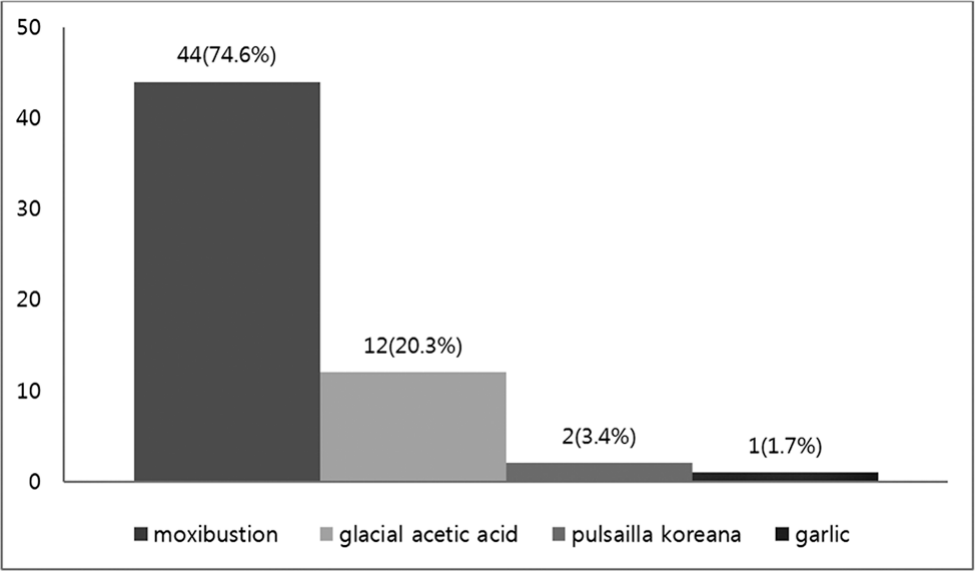
Substance of folk remedies caused burn.

The types of folk remedies that risk burn injuries include: moxibustion, glacial acetic acid, garlic substances, and herbal extracts ( *Pulsatilla koreana* ). Moxibustion (74.6%) was the most common, followed by glacial acetic acid (20.3%) ( Fig. 1 ).

Survey results for the clinical characteristics of the burns due to folk remedies are presented in Table 2 . Most patients had more than one wound and most burns were classified as severe. Therefore, the rate of surgical treatment was high (72.5%). The most common site of injury was the lower extremity including the foot.

**Table 2:** Characteristics of burn wound

Characteristics	*N* (%)
Lesion of burn a	
Head and neck	8 (12.5)
Face	3
Neck	5
Upper extremity	14 (21.8)
Shoulder	3
Elbow	4
Forearm	2
Hand (wrist, fingers)	5
Trunk	8(12.5)
Back	3
Umbilicus	5
Lower extremity	34 (53.1)
Knee	7
Lower leg	9
Ankle	6
Foot	12
Number of wounds	
Single	11 (18.6)
Multiple	48 (81.4)
Type of treatment	
Surgery	43 (72.9)
Secondary healing	16 (27.1)

a The number of patient is counted more than once if there are wounds in different parts of body.

The pathophysiology of the burn was based on the substance used in the folk remedy. Burns due to moxibustion were classified as contact burns, and burns due to glacial acetic acid, garlic, and herbal extracts were chemical burns.

### Moxibustion

**Fig. 2:**
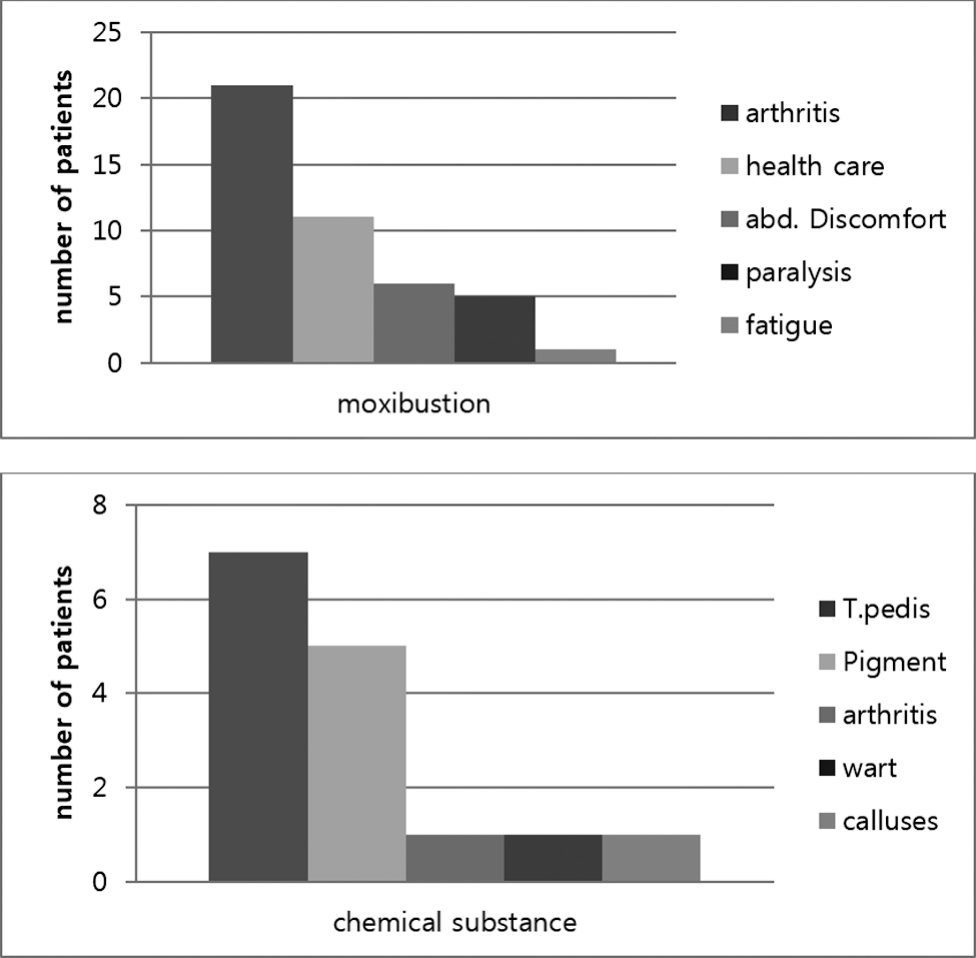
Reasons for use of each substance.

**Fig. 3:**
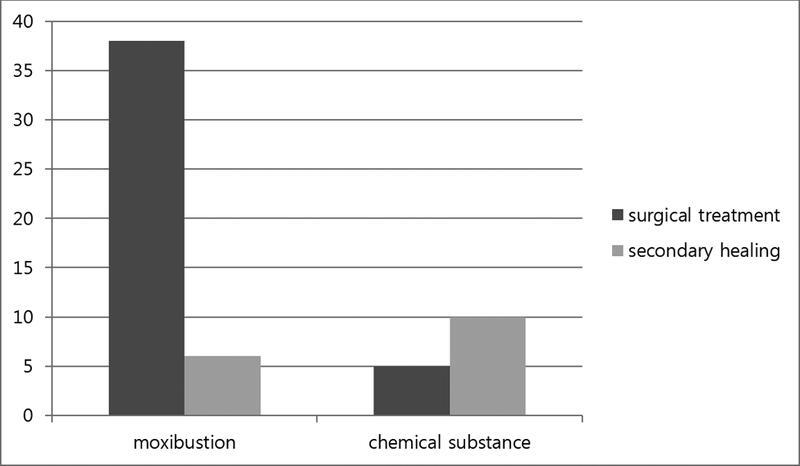
Type of treatment of each substance.

**Fig. 4:**
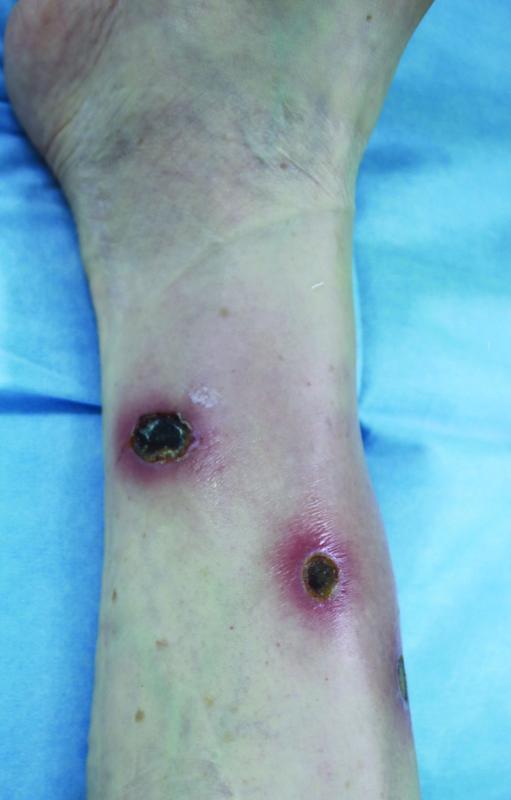
The characteristic appearance of burns due to moxibustion. Small size but multiple wounds. Round to oval shape. Black eschar and dry wound bed estimated third-degree burn.

Of the 44 patients with moxibustion burns, 33 were ≥ 60 years old (75%) and 11 were < 60 years old. Most patients had used the remedy for joint pain relief (47.7%) and health improvement (25%) ( Fig. 2 ), and the burn wounds were mainly located in joint areas. The size of the burn wounds was small (mean size, 7.81 cm ^2^ ), and the wounds were primarily round or oval, with a similar length and width. Since most of the burns were severe (excluding 11% of patients) most patients underwent surgical treatment ( Figs. 3 and 4 ).

### Chemical Substances

**Fig. 5:**
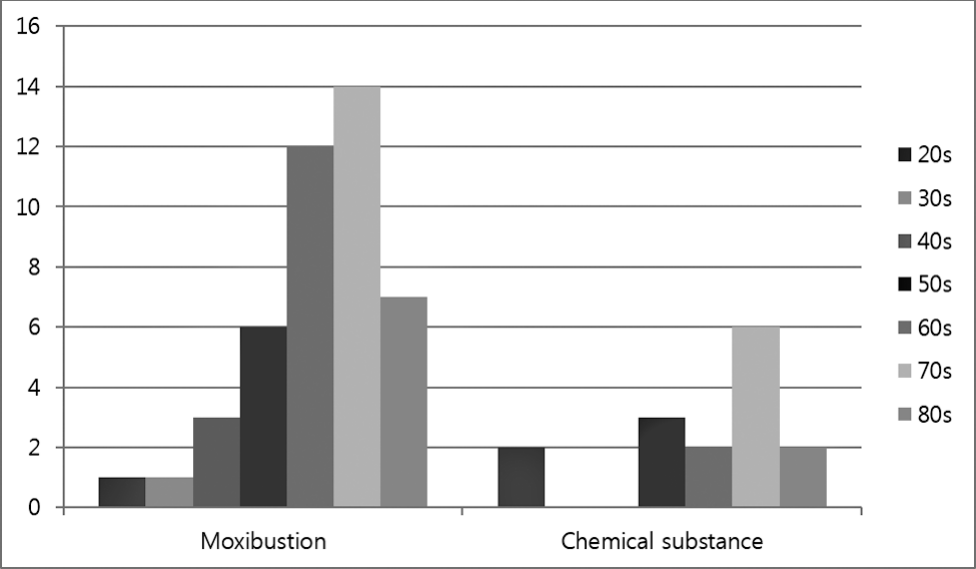
Age distribution of each substance.

**Fig. 6:**
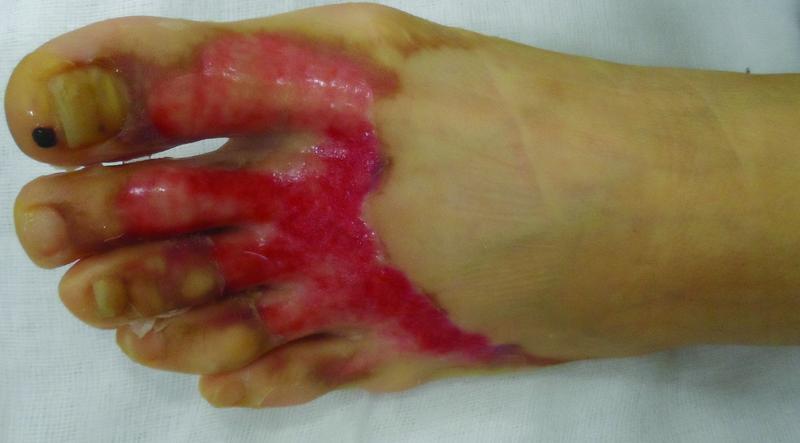
The characteristics appearance of burns due to glacial acid. Geographic shape like a flowing water. Pinkish, edematous, and wet wound bed.

Of the 15 patients with chemical burns, 40% were in their 70s, with a similar distribution among other age groups ( Fig. 5 ). Glacial acetic acid was the main substance that caused burn injuries. Glacial acetic acid was used as a folk remedy to cure tinea pedis (46.7%) or to remove a pigmented lesion ( Fig. 2 ). Although most burn lesions were on the feet, the use of glacial acetic acid to remove pigmented spots was usually on an exposed part of the body like the face or forearm. Only five patients (33.3%) underwent surgical treatment for chemical burns. Most of the patients with chemical burns healed secondarily ( Fig. 3 ). The wound due to chemical burns were characterized by irregular and wide. The wound bed had a pale or pinkish color, was edematous and wet than the around, and turned a blanch to touch ( Fig. 6 ).

## Discussion

Folk remedies, also known as traditional medicine, are comprised of traditional medical knowledge that has developed over generations, before the era of modern medicine, and is influenced by the folk beliefs of indigenous societies. Advances in science and medicine have proven that many such methods are unfounded or that they can cause serious complications. Nevertheless, some practices and medicines are still widely used. One reason for their continued use may be the fact that modern medicine does not always cure the disease. In Korea, people still use folk remedies for various reasons. According to this study, the folk remedies with a risk of burns were moxibustion, glacial acetic acid, and the use of medicinal plants such as garlic.

Moxibustion is based on traditional Chinese medicine and has been used for 2,500 years in East Asia. Moxibustion is believed to work through heat stimulation and the pharmacological effects of moxa. Moxa is made with dried *Artemisia vulgaris* . Burning moxa stimulates acupuncture points and the meridian system with the intention of promoting nourishing warmth. 1 2 Moxibustion effects is thought to including the thermal effect, radiation effects, and pharmacological effects of moxa. The thermal effects cause pigmentation, infection, and mainly burn complication. The other radiation effects and pharmacological effects could be systemic side effects. According to the papers reported, systemic side effects were allergies, cough, nausea and vomiting, fetal harm, basal cell cancer, and death. Most common systemic side effect was allergy. Analysis of case in reports, fetal harm, basal cell cancer, and death are suggested not a side effect of moxibustion, but rather an inappropriate treatment. 3 4

The moxibustion technique involves placing burning moxa material on the patient's skin, directly or indirectly. Once the temperature of the skin surface rises to 65°C, tissue damage can result in burns, although the degree of the burn varies depending on the duration of the moxibustion treatment and the patient's posttreatment condition. 1 2

The most common burn lesions were in joint areas (e.g., ankle, knee, elbow) because the most common reason for moxibustion was relief of joint pain, and because joints are considered meridian points. Because heat was transmitted directly through the skin, the moxibustion burns were mostly severe and treated with surgery. A previous report on moxibustion in Korea showed similar results. 2 Moxibustion burns account for the highest percentage (68.6%) of burns due to folk remedies. In Korea, moxibustion is regarded as an alternative medicine rather than a folk remedy because people believe in the principles of Oriental medicine (i.e., Eastern medicine or traditional Chinese medicine) such as acupuncture points or *Qi* . In addition, people believe that the more scarring produced, the better the effect, and that burns are not serious injuries. 1 For this reason, moxibustion was the most common source of folk remedy burns.

Glacial acetic acid is a weak, colorless, and anhydrous (undiluted or free of water) form of acetic acid and is classified as a weak acid. Diluted glacial acetic acid is suitable for use in the plastics and food industries. A diluted solution of acetic acid is known as vinegar, ethanoic acid, or ethylic acid. However, this weak acid is strong enough to mildly corrode metals such as iron, magnesium, and zinc; thus, it also poses a risk for burns when placed in prolonged contact with the skin. The mechanism of a chemical burn is different from that of a thermal burn. Chemicals can remain in the tissue and cause progressive damage. Specifically, acids cause consolidation of loose connective tissue, thrombosis of blood vessels, ulceration, fibrosis, and hemolysis of red blood cells, which leads to the production of eschar and necrosis. In mild cases, only keratin desquamation or superficial bullae may occur.

Glacial acetic acid is used as a folk remedy to cure tinea pedis and warts, and for the removal of pigmented lesions (e.g., nevus, freckle, solar lentigo). This false belief arose from the phenomenon of skin desquamation by the dissolution action of acids. Burns due to glacial acetic acid have also been found in other cultures, according to a report by Doles et al 5 on the accidental topical applications of improper dilutions of glacial acetic acid during medical practice. In contrast, in Korea, glacial acetic acid burns were the result of deliberate use as a folk remedy. 6

The recognized medicinal plants that have been used as folk remedy medicines since ancient times are pepper ( *Piper* spp., Piperaceae), mustard ( *Sinapis* spp., Brassicaceae), fig ( *Ficus carica* , Moraceae), walnut ( *Juglans regia* , Juglandaceae), corn buttercup ( *Ranunculus arvensis* , Ranunculaceae), and garlic ( *Allium sativum L* ., Alliaceae). 7

Several reports of burn complications from folk remedies have been garlic burns. Garlic ( *Allium sativum* ) has been used as a natural medicine for various diseases in various cultures since 3000 BC. Garlic was used as an antibiotic for the prevention of wound infection during the First and Second World Wars. 8 Allicin is a garlic compound that has demonstrated antibacterial and antifungal properties. Allicin is also thought to activate fibroblasts, and thus potentially accelerate wound healing. 9 A systematic review of English language journals found 32 papers reporting 39 cases of garlic burns. 7 There have also been case reports of burns caused by garlic in non-English journals, including a case report in Korea. 8

In Korea, some reported burns were caused by mustard and the Korean pasque flower ( *Pulsatilla koreana* ). The Korean pasque flower is a native herb used in specific folk remedies for arthritis. 10 11

Studies have reported reasons for the continued use of folk remedies such as hospital costs, access to hospitals, and low sociocultural status. 12 Although hospitals are easy to access in Korea and the hospital costs are relatively low, folk remedies are still used. While it is easy to access professional information online, it is also easy to find incorrect information on inappropriate and unscientific methods of treating diseases and injuries (e.g., “how to cure tinea pedis,” “how to improve joint pain”). People may be convinced to trust and imitate the “traditional folk remedies” found on the Internet.

The papers published so far were clinical presentations by complication of each folk material. But, the author collected and reported various ingredients of folk remedies that could produce burns. The reason why each folk remedy is used and the mode of burns and characteristics of burns are reported.

A limitation of this study was that the results were derived from analyzing the data of patients treated at a single center.

Folk remedies are widely used, even though they may be ineffective and have the potential for serious burns. There is often limited awareness among the public of the risks and lack of treatment efficacy. The purpose of this article was to provide epidemiological information on the clinical aspects of folk remedy-induced burns and the severity of folk remedy-induced burns, as well as to raise public awareness of the same.
